# Preparation, Characterization and In Vitro Anticancer Activity of Sulforaphene-Loaded Solid Lipid Nanoparticles

**DOI:** 10.3390/foods13233898

**Published:** 2024-12-03

**Authors:** Lijuan Han, Xiaobo Ma, Mingwen Chen, Junbo He, Weinong Zhang

**Affiliations:** 1 Key Laboratory for Deep Processing of Major Grain and Oil, Ministry of Education, Wuhan Polytechnic University, Wuhan 430023, China; mxiaobo999@163.com (X.M.); junb112he@whpu.edu.cn (J.H.); zhangweinong@163.com (W.Z.); 2 Hubei Key Laboratory for Processing and Transformation of Agricultural Products, Wuhan Polytechnic University, Wuhan 430023, China

**Keywords:** sulforaphene, solid lipid nanoparticles, characterization, stability, in vitro anticancer activity

## Abstract

Sulforaphane (SFE) extracted from radish seeds has garnered significant research attention in recent years due to its notable biological activities, particularly its anticancer properties. However, SFE is highly sensitive to the environment; therefore, solid lipid nanoparticles (SLNs) were used to embed SFE to enhance its stability. SFE-SLNs were characterized and compared with free SFE to assess the impact of SLNs on SFE. The SFE-SLNs exhibited a spherical shape with a uniform and stable distribution. FTIR analysis suggested that SLNs might distribute SFE both within and on their surface. The SLNs effectively protected free SFE from breaking down at high temperatures, in water with pH levels between 2.0 and 9.0, and while being stored for over 8 weeks at 25 °C. In addition, the SFE in SFE-SLNs exhibited a sustained release compared to a sudden release of free SFE, leading to enhanced absorption in the intestine and improved bioavailability. Embedding SFE in SLNs did not make it less effective at killing cancer cells. This study provides an effective approach to improving the efficiency and stability of SFE, which could aid in incorporating its beneficial characteristics into products such as beverages, dairy products, solid formulations, and dietary supplements.

## 1. Introduction

**Figure 1:**
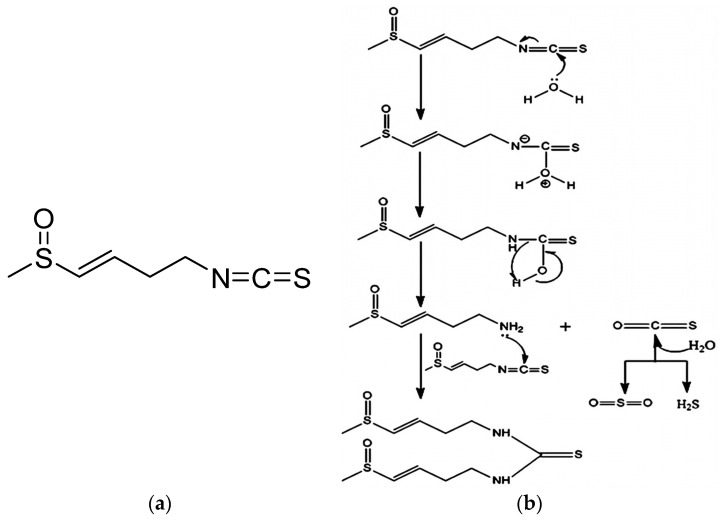
(**a**) Sulforaphene (SFE) structural formula. (**b**) Degradation pathways of sulforaphene (SFE) in water.

Isothiocyanates, byproducts of the hydrolytic breakdown of glucosinolates, are sulfur-containing chemicals present in several kinds of plants belonging to the *Brassicaceae* family, such as radish (*Raphanus sativus* L.), broccoli, kale, cauliflower, and Chinese cabbage [1]. Radish seeds contain two prominent isothiocyanates: sulforaphene (SFE, 4-isothiocyanato-1-(methylsulfinyl)-1-butene) and sulforaphane (SFA, 1-isothiocyanato-4-[(R)-methanesulfinyl] butane). SFE is a small molecular compound, as shown in Figure 1 [1], and possesses a variety of beneficial health effects, including anticancer, antioxidant and anti-mutagenic properties [2,3]. Its anticancer mechanism is to inhibit proliferation by activating detoxification and apoptosis, as well as inducing the expression of genes/proteins involved in cell cycle blockade [4], and to regulate the development of cancer cells by blocking the cell cycle and promoting apoptosis [5]. Compared with SFA, which is found at high levels in cauliflower, SFE has one more unsaturated double bond on the hydrocarbon chain than SFA, causing the activity of SFE to be 1.3–1.5 times higher than SFA [6]. Therefore, SFE can be added to food as an ingredient or used as a medicine to treat and prevent certain diseases. However, SFE is very unstable and is easily degraded in water, high-temperature conditions, alkaline conditions and various solvents [1,7]. -OH and OH- can attack the active carbon atoms in SFE -N=C=S to form a dimer for degradation (as shown in Figure 1) [8]. In addition, OH- can also react with sulfoxide groups in SFE molecules to form -CH_3_SOOH and cause degradation [9]. Because of the instability of SFE, its extraction and application are limited.

Making SFE microcapsules with natural biopolymers is also a feasible method to improve SFE stability [10]. SFE microcapsules using hydroxypropyl-beta-cyclodextrin, maltodextrin and isolated soybean protein as wall materials could effectively improve its stability against heat. In short, the easily degradable nature of SFE makes it unsuitable for medical preparations, and the degradation products may have biological side effects. Thus, improving SFE stability requires further research.

Solid lipid nanoparticle (SLN) is a new carrier developed on the basis of traditional liposomes and emulsifiers, with uniform size, large drug loading [11], relatively simple production, and the ability to protect unstable drug particles and control drug release [12,13]. Additionally, it has the advantages of good biocompatibility [14], low toxicity, simultaneous loading of hydrophilic and hydrophobic drugs and prolongation of internal circulation, and is suitable for use as a drug carrier [15,16]. SLNs are colloidal particles of submicron size, with a diameter between 50 and 1000 nm [13]. They consist of a stabilized polymer carrier consisting of a lipid matrix solid, a surfactant and some co-surfactants [17]. The location of the encapsulated compounds within the SLN depends on their chemical properties [18]. Protein-covering solid lipid nanoparticles are novel among nanocarrier systems and exhibit some advantages such as reduced particle size, elevated surface charge, good protection for hydrophobic compounds and substitution of synthetic emulsifiers by natural ones, which makes this a potential and versatile delivery system for food and pharmaceutical applications [15]. SLNs have a series of potential innovative approaches and commercial applications [19,20].

Researchers have conducted numerous studies to reduce the water and/or protic solvent content or improve the thermal stability of SFE by adding antioxidants, providing nitrogen protection, or creating SFE microcapsules using natural biopolymers [1]. Zambrano et al. [21] used water-in-oil emulsification to microencapsulate SFE, achieving high encapsulation efficiency and improving the thermal stability of SFE in aqueous solution. Lim et al. [22] extracted SFE from radish seeds and conducted proliferation inhibition experiments on A549 cancer cells, discovering its anti-proliferative properties as well as proving it to be a potent inducer of cell apoptosis. To our knowledge, no study has documented the stabilization of SFE using SLNs. Therefore, the goal of the present research was to design and characterize SFE-loaded SLNs and evaluate how well they protected against heat, pH variation, long-term storage, in vitro release, in vitro antioxidant activity, and anticancer activity compared to free SFE.

## 2. Materials and Methods

### 2.1. Materials

SFE standard (>98% pure) was purchased from Chengdu Desite Biotechnology Co., Ltd. (Chengdu, China). Radish (*Raphanus sativus* L.) seeds were obtained from Wuhan Vegetable Research Institute. Glyceryl monostearate (GMS) was purchased from Shanpu Chemical Co., Ltd. (Shanghai, China). Propylene glycol monopalmitate (PGMP) was produced internally in our laboratory. Sodium caseinate (NaCas) was purchased from TCI Development Co., Ltd. (Shanghai, China). Acetonitrile (chromatographic grade) was purchased from Tianjin kermel chemical reagent Co., Ltd. (Tianjin, China). Pepsi and pancreatin were obtained from Beijing Solarbio Science Technology Co., Ltd. (Beijing, China). DMEM/F-12 (SH30023.01B) medium, DMEM High glucose (1×) (SH30022.01B), MEM/EBSS (SH30022.01B), and Modified RPMI-1640 medium were all purchased from Hyclone Co., Ltd. Fetal Bovine Serum (FBS) was purchased from Hangzhou Si Ji Qing Biological Engineering Material Co., Ltd. (Hangzhou, China). In addition, 0.25% trypsin was purchased from Zhejiang Senrui Biotechnology Co., Ltd. (Huzhou, China); CCK8 (Cell Counting Kit-8) was purchased from BioSharp Co., Ltd. (Hefei, China); and the human lung cancer cell line A549 was obtained from Nanjing Cobioer Biosciences Co., Ltd. (Nanjing, China).

### 2.2. Preparation of SFE Crude Extract (Free SFE)

Radish seeds were pulverized using a high-speed crusher, and then sieved through a 60-mesh sieve. Distilled water was added at a solid–liquid ratio of 1:10 (*w*/*v*), and the mixture was subjected to constant temperature oscillation at 25 °C for 3 min, followed by centrifugation at 4500 rpm for 10 min. The supernatant was collected, and an equal volume of dichloromethane was added. The mixture was subjected to constant temperature oscillation at 25 °C for 30 min, followed by centrifugation at 3000 rpm for 3 min. The organic phase was collected, and dichloromethane was removed by rotary evaporation, yielding the SFE crude extract (purity 75.3 ± 0.2%).

### 2.3. SFE Content Detection

We employed a C18 column (250 mm × 4.6 mm × 5 μm), with ultrapure water and acetonitrile as the mobile phase, using acetonitrile from 20% to 60% for 15 min, then from 60% to 100% for 5 min. We used a UV detector detection wavelength of 254 nm, a flow rate of 1 mL/min, and a sample volume of 10 μL.

The appropriate amount of SFE standard substance was dissolved in acetonitrile, and an SFE standard reserve solution with a concentration of 10.0 mg/mL was prepared, which was diluted to series concentrations of 1.0, 0.5, 0.2, 0.1, 0.05, 0.02, 0.01, 0.005 and 0.002 mg/mL. The prepared SFE standard solutions were detected by HPLC, and the corresponding peak area was recorded. The standard curve was plotted with the injection concentration of SFE as the abscissa and the corresponding peak area as the ordinate. The linear regression equation is y = 9571.4x − 9.3704 (R^2^ = 0.9991). Stated differently, the linear relationship is good when the concentration is 0.002~1.0 mg /mL.

### 2.4. Preparation of SFE-SLNs

#### 2.4.1. Production of SFE-SLNs

A total of 80 mg of lipids containing the mixture of PGMP and GMS at a molar ratio of 1:1 was melted at 65 °C under continuous stirring. To produce SFE-SLNs with varying theoretical loading efficiency (TLE), a specific quantity of SFE crude extract was added and stirred at 65 °C for 10 min. Then, 30 mL 5 mg/mL NaCas solution was added and stirred at 65 °C for 20 min, followed by 7 min of ultrasonic treatment. Finally, freeze drying was carried out.

#### 2.4.2. SFE Loading Parameters

Samples with TLEs of 4%, 10%, 16%, 22%, and 28% were selected for subsequent evaluation of the actual loading efficiency (ALE), entrapment efficiency (EE), stability, and sustained release. To calculate the TLE (%) and ALE (%) of SFE-loaded SLNs, the following equations were applied:

(1) $$\mathrm{TLE}\;(\%)=\frac{Mass\left(g\right)\;of\;input\;SFE\;(based\;on\;the\;purity\;of\;SFE\;crude\;extract)}{Mass\left(g\right)\;of\;weighted\;lipids\;mixture}\times 100$$

(2) $$\mathrm{ALE}\;(\%)=\frac{Mass\left(g\right)\;of\;actual\;detected\;SFE\;in\;SFE\;-\;SLNs}{Mass\left(g\right)\;of\;total\;weighted\;lipids\;mixture}\times 100$$

The ultrafiltration method was carried out for different entrapment efficiencies (%). The EE was determined as shown in Equation (3):

(3) $$\mathrm{EE}\;(\%)=\frac{Mass\left(g\right)\;of\;loaded\;SFE\;in\;SLNs}{Mass\left(g\right)\;of\;input\;SFE}\times 100$$

### 2.5. Characterization of SLNs

#### 2.5.1. Particle Size, Polydispersity Index and Zeta Potential

The mean particle size (nm) and polydispersity index (PDI) were determined using Zetasizer Nano ZS90 (PerkinElmer, Worcestershire, UK), equipped with a particle size range of 2 nm to 3 μm. The analyzed samples were diluted 30 times in ddH_2_O and the PDI value was tested three times during one cycle, with 33 runs in each measurement. The cumulative approach was employed for data analysis. Zeta potential analysis was performed using Zetasizer Nano ZS90 (PerkinElmer, UK). The samples were analyzed without dilution. The values are reported as the average of three repeated measurements per sample. The data are expressed as the arithmetical mean ± standard deviation.

#### 2.5.2. Transmission Electron Microscope (TEM)

The TEM analysis was conducted utilizing the HT7700 instrument (Hitachi Ltd., Mito, Japan). One drop of bare SLN or SFE-SLN dispersion (an aqueous solution of SLN) was placed on the copper grid for 3~5 min, and then negatively stained with one drop of a 2% (weight/volume) aqueous solution of phosphotungstrate acid for contrast enhancement. The excess solution was absorbed by filter paper, and the samples were allowed to dry before analysis.

#### 2.5.3. Fourier Infrared Spectroscopy (FTIR)

FTIR analysis was carried out using an FT-IR spectrometer (Frontier, PerkinElmer, UK). We applied a suitable quantity of SFE crude extract sample directly onto compressed potassium bromide pellets and conducted infrared spectroscopy scanning. NaCas, PGMP, GMS, the SFE-loading sample, and blank SLNs were carefully and uniformly ground with potassium bromide powder, and subsequently compressed into pellets. Each KBr pellet was scanned over a wave number region of 4000 to 400 cm^−1^.

### 2.6. Stability Assessment

Thermal stability, pH stability, and long-term stability studies were conducted to evaluate the stabilizing effect of SLN on SFE.

A certain mass of SFE crude extract and SFE-SLNs (ALE 16.17%) were placed in 10 mL glass sample bottles at 60 °C. Samples were taken every 5 h and acetonitrile was added to dissolve them during the heating time of 0 to 35 h. An HPLC assay as described earlier (see Section 2.3) was used to detect the content of SFE in SFE crude extract and SFE-SLNs, and evaluate the thermal stability of free SFE and SFE-SLNs based on their retention rate.

Buffer solution was prepared with 0.2 mol/L Na_2_HPO_4_ solution and 0.1 mol/L citric acid solution with pH values from 2, 3, 4, 5, 6, 7, 8 to 9 according to the standard. The SFE and SFE-SLNs of a certain mass were added and dissolved in buffer solution with different pH values. The SFE content was analyzed and the SFE residual rate was calculated.

The samples were stored at 25 °C for 8 weeks under sealed conditions and sampled once a week to evaluate the long-term stability. The SFE content was detected by HPLC, and the SFE residual rate was calculated. Each sample was analyzed three times.

### 2.7. In Vitro Release Study

The kinetic release profile of SFE and SFE-SLNs were evaluated under simulated gastrointestinal conditions using a dialysis membrane method. The free SFE and SFE-SLNs of a certain mass were taken, dissolved in distilled water and placed in respective dialysis bags (MWCO = 3500 Da). The dialysis bags were placed in 150 mL of simulated gastric fluid (SGF) (Pepsin 0.32%, NaCl 0.2%, pH 1.2) and incubated for 2 h in a thermostatic oscillator at 37 °C and 100 rpm. After a specified amount of time, 1 mL of release medium was withdrawn and an equal amount of fresh medium was added. SFE was analyzed using the HPLC method. After releasing for 2 h in gastric juice, the dialysis bag was transferred to the simulated intestinal fluid (SIF) (Trypsin 1 wt%, pH 6.8 PBS, 0.05 mol/L) for 4 h.

### 2.8. In Vitro Anticancer Activity

#### 2.8.1. Cell Culture

Human lung cancer cell line A549 was cultured in DMEM (Dulbecco’s Modified Eagle Medium) with high sugar (90%) and 10% FBS (Fetal Bovine Serum). The medium containing the A549 cells was cultured at a constant temperature of 37 °C with 5% CO_2_ gas in an incubator.

The culture medium was discarded, and 3–5 mL of PBS was added for washing twice. After removing the PBS, 0.6 mL of trypsin was added, and the culture dish was gently shaken to ensure that the trypsin covered all the cell surfaces. Then, the cells were incubated at 37 °C in a constant-temperature CO_2_ incubator until they were detached from each other (the digestion time for this cell line was 30 to 50 s). The cells were gently tapped to avoid excessive bubble formation during the process. Then, 2 mL of complete culture medium was added to halt the digestion process and the cells were gently tapped to mix them evenly. The mixed cell suspension was divided into several portions, supplemented with an appropriate amount of culture medium, and placed in the CO_2_ incubator for further culturing. The subculturing ratio depended on the cell growth situation; typically, most cells were subcultured at a ratio of 1:2 to 1:4.

The digested cells were centrifuged to remove the supernatant, and the cells were gently resuspended by adding the prepared cryopreservation solution (the cryopreservation solution did not need to be preheated) and labeled. The formulation of the cryopreservation solution was 50% complete medium with 40% FBS and 10% DMSO, and the cryopreservation tubes were stored in liquid nitrogen tanks at 4 °C for 30 min, −20 °C for 2 h, and −80 °C overnight.

The culture medium was preheated to room temperature and approximately 5 mL of the medium was added to a sterile T-25 culture flask prepared in the laminar flow hood. We retrieved the target cells from the liquid nitrogen tank and placed them in a 37 °C water bath with agitation (1500 rpm) until completely melted. We then retrieved the cryovials, wiped them dry, and sprayed them with 75% ethanol before transferring them to the sterile area. We discarded the cryopreserved solution, resuspended the cells in 1 mL of complete culture medium, gently aspirated the cells, and added them to the prepared culture flask. The culture flask was gently swirled to evenly distribute the cells and then placed in a CO_2_ incubator for overnight culture and observation.

#### 2.8.2. Cell Activity Assay

Log-phase A549 cells were digested with trypsin and prepared into a cell suspension. The cell suspension was seeded into a 96-well plate at a density of 1 × 10^4^ cells per well, with 100 μL of cell suspension added to each well. Three replicate wells were seeded for each concentration, and the plate was placed in a 5% CO_2_, 37 °C incubator for 24 h before preparing the monolayer culture.

We dissolved a certain mass of SFE crude extract and SFE-SLNs with an ALE of 16.17% in DMSO solvent. We then prepared a certain concentration of stock solution based on the content of SFE in the sample. Corresponding to the same mass of SFE in SFE-SLNs, blank SLNs were also prepared into stock solutions of certain concentrations. We then diluted the stock solutions to 100 µmol/L, 50 µmol/L, 25 µmol/L, 12.5 µmol/L, 6.25 µmol/L, and 0 µmol/L, respectively. The original culture medium in each well of cells was discarded and replaced with a culture medium containing different SFE concentrations for treatment for 24 h, 48 h, and 72 h.

After 24 h, 48 h and 72 h, each well was replenished with 10 μL of CCK-8 solution and incubated in the incubator for 2 h. The absorbance at 450 nm was determined by an enzyme marker, and the proliferation rate was calculated according to Equation (4). The concentration of free SFE and SFE-SLNs needed to inhibit cell growth by 50% (IC_50_) was calculated from the dose response curves for each cell line [23].

Proliferation rate (%) = (Experimental group − Blank)/(Control group − Blank)(4)

#### 2.8.3. Cytomorphological Observations

Different concentrations of blank SLNs (100 μmol/L) and SFE-SLNs (6.25 μmol/L, 25 μmol/L, and 100 μmol/L) were used to treat A549 cells for 24 h, 48 h, and 72 h, respectively, and the growth status and morphological changes of the cancer cells were determined using an inverted microscope (Wirsam, Olympus CKX41, Tokyo, Japan).

### 2.9. Statistical Analysis

All experiments were repeated three times. SPSS software 19.0 (IBM SPSS statistic) was used for one-way analysis of variance (ANOVA) with Duncan’s multiple comparison test. A significance level of *p* < 0.05 was considered as statistically significant.

## 3. Results and Discussion

### 3.1. Determination of Particle Size, Zeta Potential and PDI

Table 1 summarizes the mean particle size (nm), zeta potential (mV), and polydispersity index (PDI) of SLNs with varying TLE. An ideal SFE-loaded formulation typically possesses a smaller particle size (facilitating drug absorption via the lymphatic route), a high zeta potential (essential for the formulation’s stability), and a high entrapment efficiency (ensuring effective drug loading in nanoparticles) [24]. The mean particle size and zeta potential of blank-SLNs and SFE-SLNs ranged from 113.3 nm to 129.9 nm, and from −24.1 mV to −33.0 mV, respectively. The particle size of nanoparticles and the absolute value of zeta potential decreased as the SFE TLE increased, indicating that the stability of SLNs increased after being loaded with SFE. Given that the particle size of the SFE-SLNs was less than 200 nm, it is expected that they could be readily absorbed through the lymphatic channel [24,25].

The PDI is a measure of the uniformity of particle size and serves as a crucial indicator for characterizing particle size [26]. According to Masarudin et al. [27], when considering a particular range of distribution, a smaller distribution coefficient corresponds to a more uniform particle size. Our research indicated that the PDI values of SLNs loaded with SFE were all below 0.25 and exhibited no significant difference compared to that of the blank-SLNs. This suggests that the SFE-SLNs had a uniform particle size and avoided particle agglomeration [28]. Therefore, the particle size, zeta potential and PDI showed that the system was distributed evenly and stable.

Table 1 also shows the ALE and EE of the SFE-SLNs. The ALE values of SFE-SLNs were similar to the TLE. The EE values of SFE-SLNs ranged from 31.52 to 56.94%. These results were in agreement with the findings of Laein et al. [28]. They reported that peppermint essential oil-loaded SLNs with different amounts of GMS have an EE range of approximately 38.9~55.5%. The EE decreased as the ALE increased, and the SFE-SLN with an ALE of 16.17% had a maximum EE of 56.94%. Nahr et al. [29] found that various parameters have a substantial influence on the EE values. These factors include the solubility of core materials in the lipid matrix, the compression of lipid structures, the types and concentrations of surfactant, and the ambient variables. Our findings suggest that SLNs have a restricted capacity to incorporate SFE into the lipid matrix. PGMP and GMS form a lipid mixture with connecting crystals that exhibit relatively stable properties [30]. When SFE was mixed with lipids, a fraction of SFE would enter the lattice formed by the lipid matrix. SFE would flee from the lattice when the ALE was low, and any extra SFE within the lattice would be exposed when the ALE was high.

While the efficiency of including SLNs with SFE is not particularly high, SLN delivery systems nonetheless provide protection against the degradation of SFE caused by protonic solvents or heat due to its encapsulation within solid lipids, as explained by Lammari et al. [31].

### 3.2. Transmission Electron Microscope (TEM) of SFE-SLNs

**Figure 2:**
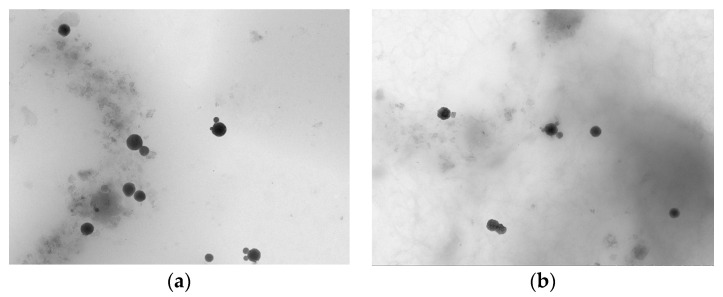
Transmission electron microscopy photograph of (**a**) blank-SLNs and (**b**) SFE-SLNs with an actual loading efficiency of 16.17%.

The morphological properties of blank-SLNs and SFE-SLNs are depicted in Figure 2. The results revealed that the shape of SLNs is spherical, with sizes ranging from 100 nm to 200 nm. The spherical shape of SLNs provides them the greatest ability for controlled release and preservation of the encapsulated SFE. The spherical shape provides the most efficient route for the movement of SFE entrapped in the nanoparticles, while also resulting in the smallest contact surface with the aqueous medium of the dispersion phase compared to other nanoparticle shapes [32].

### 3.3. Fourier Transform Infrared Spectrum (FTIR) Study

**Figure 3:**
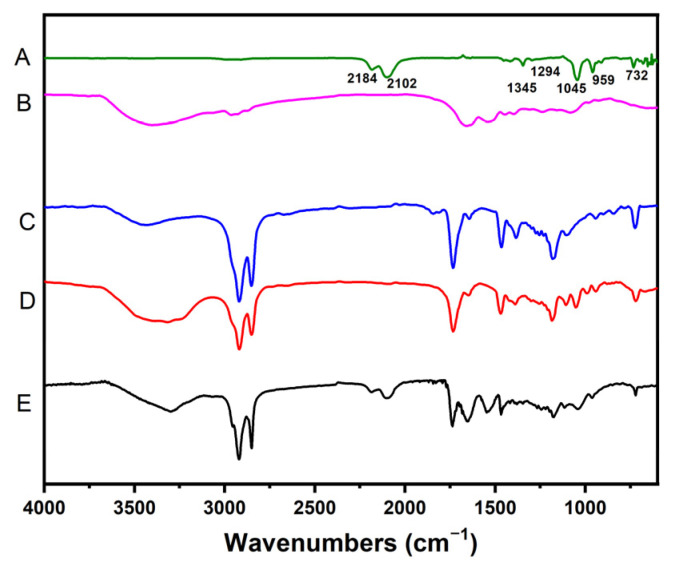
FTIR of (A) SFE, (B) NaCas, (C) PGMP, (D) GMS, and (E) SFE-SLN.

Figure 3 displays the FTIR spectra of SFE, PGMP, GMS, NaCas, and SFE-loaded SLNs. In the FTIR spectrum, SFE showed strong absorbance peaks at 2184 and 2102 cm^−1^, which were caused by N=C=S stretching vibration; peaks at 1294 and 1045 cm^−1^ were due to C-N and S=O bonds; the peak at 1345 cm^−1^ was caused by the deformation vibration of C-H from CH_3_; the absorbance at 959 cm^−1^ corresponded to the C=C bond; and that at 732 cm^−1^ was ascribed to the deformation vibration of C-H from CH_2_. The absorption at 1345 and 1294 cm^−1^ vanished in the SFE-SLN spectrum, and the intensity of other characteristic peaks of the SFE spectrum were weakened. This suggests that a certain quantity of SFE might be enclosed within the structure of the lipid matrix, while the remaining SFE was embedded on the surface of SLNs.

### 3.4. Study on Thermal Stability, pH Stability, and Long-Term Stability

**Figure 4:**
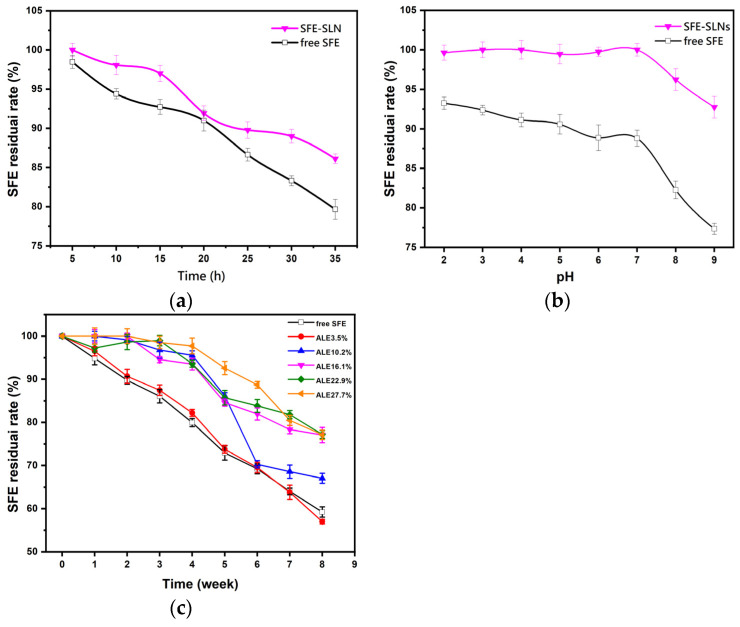
(**a**) Thermal stability of free SFE and SFE-SLNs (ALE 16.17%) at a temperature of 60 °C and (**b**) the pH stability in buffer solution with various pH values; (**c**) the long-term stability of free SFE and SFE-SLNs at a temperature of 25 °C during storage.

Thermal stability, pH stability, and long-term stability experiments were performed to assess the impact of SLN on SFE (Figure 4), with the SFE retention rate acting as an indicator. The issue of thermal stability is significant for SLNs due to the sensitivity of most bioactive compounds to high temperatures. The heat-stable SLNs may protect SFE against heat degradation. As shown in Figure 4a, SFE decreased when exposed to high temperature (60 °C) for different times due to the low SFE heat-degradation resistance. The instability of its conjugated structure and the presence of carbon atoms in the nucleophile enabled SFE to react with the nucleophile, accelerating its degradation, while the retention rate of SFE in SFE-SLNs was higher than that of the free SFE; after 35 h of incubation at 60 °C, there was still 86.11% SFE left in SFE-SLNs. Previous studies reported that an effective way to improve the stability of SFE at high temperature (60 °C) is by adding antioxidants [33]. The findings in this study suggest that SLN loading might be a novel strategy for enhancing the thermal stability of SFE.

The stability of free SFE and SFE-SLNs in aqueous solutions within the pH range of 2.0–9.0 was investigated (within 30 min after adjusting the sample pH) (Figure 4b). The degradation of free SFE in aqueous solution increased drastically, particularly under alkaline conditions. Only 77.35% of the free SFE was left at pH 9, as SFE was found to be highly sensitive to alkaline pH levels [34]. The degradation of SFE in SFE-SLNs was slower than that of free SFE. The residue rate remained close to 100% from pH 2.0 to pH 7.0, indicating that SLN effectively protected SFE from degradation. Under pH 9.0, the retention rate of SFE in SFE-SLNs increased by 17.63% compared to free SFE. However, there was still a tiny degree of degradation under alkaline circumstances, since OH- may attack the active carbon atoms of N=C=S in SFE and form a dimer to degrade. Therefore, when patients are using SFE-SLNs, they should be careful not to take them combined with alkaline food or medicine. Even in a short amount of time, SFE may undergo partial deterioration. It should be consumed in a non-alkaline environment, as far as possible, to achieve optimal effectiveness.

The results of the long-term stability study of free SFE and SFE-SLNs are shown in Figure 4c. After being stored at 25 °C for 8 weeks, the residual rate of free SFE was 59.22%, similar to that of SFE-SLNs with an ALE of 3.5%. The storage stability of SFE in SFE-SLNs showed a notable improvement when the ALE exceeded 10%, with a residual rate of 77.12% after 8 weeks of storage. SFE could leak out of the lipid matrix-formed lattice when the ALE of SLNs was small, resulting in no improvement in SFE stability. When the ALE increased, SFE was able to enter the lattice better, thereby improving its stability. Previous studies indicated that an equal molar ratio of PGMP and GMS was the most effective mixture for retaining α-form crystals, which had a synergistic effect on the crystal formation [35]; and the emulsifier NaCas on the outer layer can encapsulate SFE once it enters the lattice. Therefore, the combination of SFE, the lipid mixture matrix, and the emulsifier improved SFE stability when the ALE increased.

### 3.5. In Vitro Release Study

**Figure 5:**
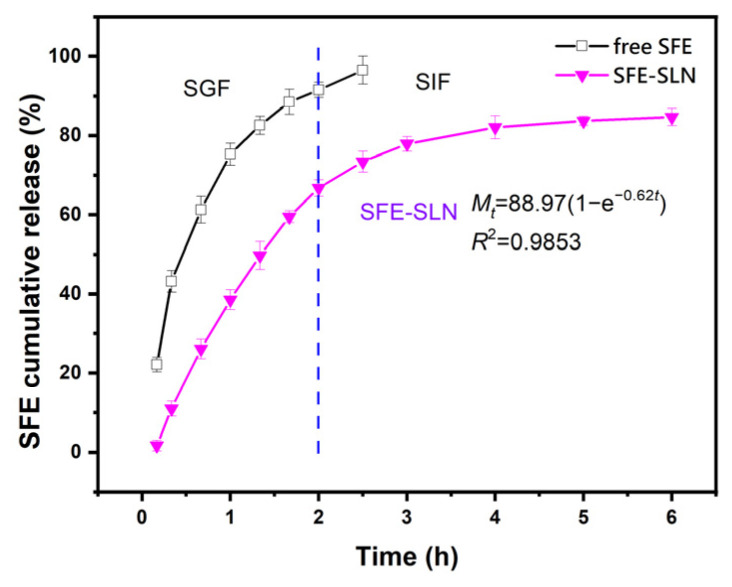
In vitro release profile of free SFE and SFE-SLNs (ALE 16.17%) in SGF and SIF.

In vitro drug release is an important indicator for evaluating drug quality. In order to investigate the in vitro release patterns of SFE in free SFE and SFE-SLNs, forward dynamic dialysis was used to simulate the release of SFE under gastric and intestinal conditions. We evaluated the release rate of SFE by detecting the cumulative release of SFE over a certain period of time. As shown in Figure 5, following digestion by gastric juice, the cumulative release rate of free SFE was 91.57%, whereas only 66.76% was observed for SFE-SLNs. After a nonlinear fitting, the release profiles of SFE in SFE-SLNs showed an exponential function with a correlation coefficient of 0.9853, indicating the strong relationship between release rate and time. After a 0.5 h digestion with intestinal fluid, the cumulative release rate of SFE in free SFE reached 96.55%, while it was only 73.42% in SFE-SLNs at this time. After a 6 h digestion in gastrointestinal fluid, the cumulative release rate of SFE in SFE-SLNs was 84.65%. These results indicate that SLNs effectively slow down the release of SFE in the digestive tract, leading to enhanced absorption in the intestine and improved bioavailability. The sustained release of SFE in SFE-SLNs can reduce the frequency of administration and minimize side effects [36]. On the other hand, the cumulative release curve of SFE-SLNs indirectly confirmed the previous FTIR-based speculation that SFE was distributed within the lattice and crystal surface of the lipid matrix in SLNs. The SFE embedded on the surface of SLN crystals was first released during digestion in the stomach, while due to the encapsulation protection of SLNs, the SFE in the lipid matrix lattice was slowly released after entering the intestine.

### 3.6. In Vitro Anticancer Activity of SFE-SLNs Against A549 Cells

**Figure 6:**
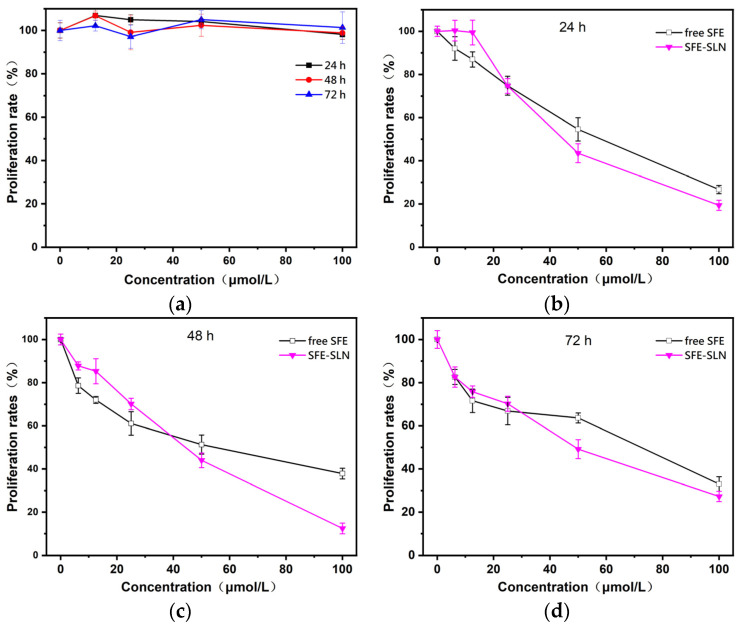
(**a**) Effect of blank-SLNs on proliferation rate of A549 cells. Effects of free SFE and SFE-SLNs on proliferation rate of A549 cells after incubation for different times ((**b**) 24 h; (**c**) 48 h; (**d**) 72 h) at various concentrations (0 µmol/L, 6.25 µmol/L, 12.5 µmol/L, 25 µmol/L, 50 µmol/L, and 100 µmol/L).

SFE is a natural bioactive ingredient that has no cytotoxicity to normal cells [37]. However, SFE can promote apoptosis in human cancer cells by disrupting mitochondrial membrane potential and blocking the cell cycle [38]. Given SFE’s excellent anticancer performance, it is necessary to determine whether SLN loading will affect SFE’s in vitro anticancer activity. The in vitro inhibition experiments were carried out on A549 cells with blank-SLNs, free SFE and SFE-SLNs, and the proliferation curves of A549 cells were obtained after incubation for different times at different concentrations. Figure 6a demonstrates that blank-SLNs exhibited no toxicity towards A549 cells. The cell viability remained consistently around 100% across all concentrations (ranging from 12.5 μmol/L to 100 μmol/L) for 24 h, 48 h, and 72 h. This could be attributed to the fact that the constituents of the SLNs are physiologically compatible and have low toxicity [39]. Moreover, these components exhibit a specific inhibitory impact on cancer cells only when present in high quantities. The low micromolar dose used in this investigation did not result in any inhibitory effects on cancer cells.

The human lung cancer A549 cells were exposed to various concentrations of the free SFE and SFE-SLNs for 24 h, 48 h, and 72 h. Figure 6b–d show that when the concentrations of free SFE and SFE-SLNs were raised or the incubation time was extended, the free SFE and SFE-SLNs had a significant inhibitory effect on the A549 cells. When the A549 cells were exposed to 6.25 μmol/L SFE-SLNs for 24 h, 48 h, and 72 h, their proliferation rates were 8.32%, 9.10%, and 0.07% lower than when they were exposed to free SFE. On the other hand, A549 cells that were incubated with 100 μmol/L SFE-SLNs for 24 h, 48 h, and 72 h had proliferation rates that were 7.32%, 25.44%, and 5.78% lower than when they were exposed to free SFE. These findings indicate that at lower concentrations, free SFE exhibited stronger inhibitory effects on A549 cells compared to SFE-SLNs. Nevertheless, at doses beyond 25 μmol/L, SFE-SLNs consistently exhibited superior inhibitory effects on A549 cells compared to free SFE.

**Figure 7:**
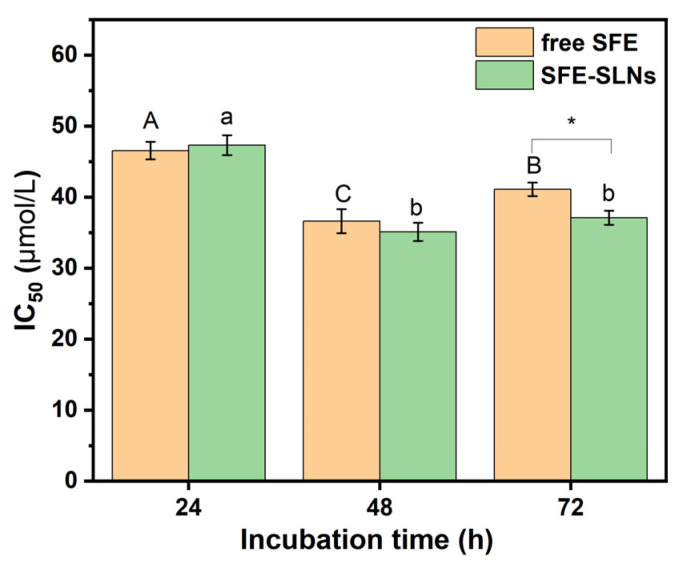
The IC_50_ value of free SFE and SFE-SLNs on A549 cells at 24 h, 48 h and 72 h. A–C, a–c, and *: Different letters represent significant differences (*p* < 0.05).

To determine the cell proliferation, the CCK8 method was used and the IC_50_ value was determined (Figure 7). The IC_50_ values of the A549 cancer cells exposed to free SFE and SFE-SLNs were decreased with the prolongation of culture time. The IC_50_ value of free SFE inhibition on A549 cells in this study was similar to values reported in previous studies and within the normal range [22]. In addition, except for 72 h of treatment, the IC_50_ value of free SFE was close to that of SFE-SLNs.

The analysis of the proliferation rate of the A549 cells and IC_50_ values demonstrates that embedding SFE in SLNs did not weaken its apoptotic effect on cancer cells. In other words, SFE-SLNs did not weaken SFE’s inhibitory efficacy.

### 3.7. Morphological Observation of the Nucleus of Cancer Cells After Subjecting to SFE-SLNs

**Figure 8:**
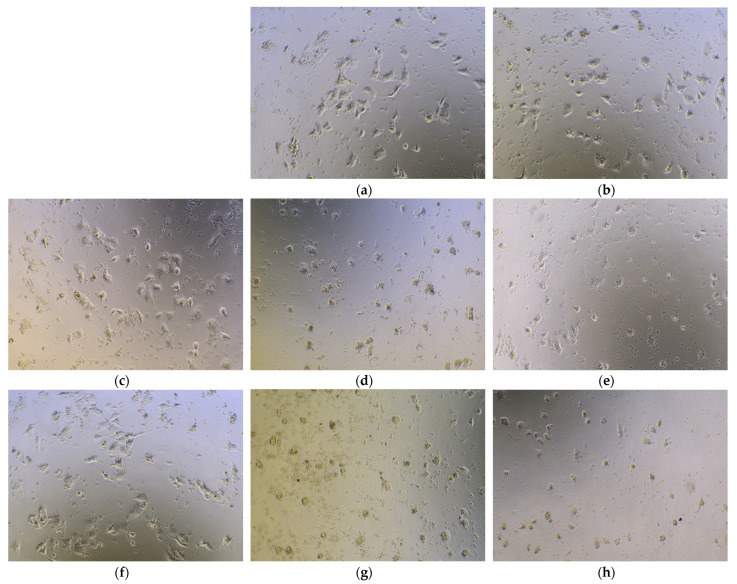
Morphological changes in A549 cells: (**a**) viable cells without drug treatment; (**b**) cells subjected to 100 μmol/L blank-SLN for 24 h; (**c**,**e**,**g**) cells incubated for 24 h and (**d**,**f**,**h**) 72 h with SFE-SLNs at 6.25 μmol/L (**c**,**d**), 25 μmol/L (**e**,**f**), and 100 μmol/L (**g**,**h**).

The effects of blank SLN and SFE-SLNs on the cell morphology of A549 are shown in Figure 8. When compared with the viable A549 cells (Figure 8a), subjecting the cells to 100 μmol/L of blank SLN did not alter the growth status and the morphology of the cells. All the cells exhibited a normal appearance, with vigorous cell activity, good condition, good luster, and strong vitality, which is in line with the finding in Figure 6a that blank SLNs had no apoptotic effect on A549 cells.

The variations in morphology seen in Figure 8c–h indicate the substantial inhibitory effect of SFE-SLNs on A549 cells. Figure 8c shows that SFE-SLNs had a significant inhibitory effect on A549 cells, even at low concentrations (6.25 μmol/L). The cells exhibit wrinkling and decreased glossiness. At a high concentration (Figure 8g), the cells exhibit pronounced wrinkling, low activity, and a significant decrease in cell count. Most cells shrink, with a large number undergoing apoptosis. With an increase in the concentration of SFE-SLNs and a longer exposure duration (Figure 8g,h), the A549 cells underwent a transition from a plump appearance to a constricted state, gradually detaching from each other. Cell viability decreased progressively, with some cells starting to disintegrate into fragments. Granules within the cytoplasm increased gradually, indicating apoptosis or necrosis of the cells. Comparing Figure 8b and g, it is evident that the morphologies of cells treated with SFE-SLNs and blank-SLNs differ significantly, consistent with previous results on cell viability. Therefore, encapsulating the SFE into SFE-SLNs does not diminish its overall anticancer effect.

## 4. Conclusions

In the current study, SLNs successfully embedded free SFE extracted from radish seeds. The SFE-SLNs exhibited a spherical shape with a uniform and stable distribution. Nanoparticle encapsulation could effectively protect free SFE from breaking down at high temperatures, in water with pH levels between 2.0 and 9.0, and while being stored for a long time. SFE might be both enclosed within and on the surface of SLNs. Importantly, SLNs could effectively slow down SFE’s release in the digestive tract, leading to improved absorption in the intestine and bioavailability. And embedding SFE in SLNs did not weaken its inhibitory effect on A549 cancer cells, according to the analysis of the A549 cell proliferation rate and IC_50_ values. This study provides an effective approach to improving the efficiency and stability of SFE, which could aid in incorporating its beneficial characteristics into products such as beverages, dairy products, solid formulations, and dietary supplements.

## Figures and Tables

**Table 1:** Determination of mean particle size (nm), zeta potential (mV), PDI, actual loading efficiency (%ALE), and entrapment efficiency (%EE) of SLNs with different theoretical loading efficiency (%TLE).

TLE (%)	Particle Size (nm)	Zeta Potential (mV)	PDI	ALE (%)	EE (%)
0	129.9 ± 1.0 ^a^	−32.4 ± 0.8 ^a^	0.219 ± 0.007 ^a^	3.50 ± 0.04 ^e^	32.85 ± 2.58 ^c^
4	127.7 ± 1.5 ^a^	−29.6 ± 1.5 ^b^	0.210 ± 0.012 ^a^	10.25 ± 0.72 ^d^	40.62 ± 2.79 ^b^
10	125.4 ± 1.4 ^b^	−33.0 ± 1.2 ^a^	0.214 ± 0.006 ^a^	16.17 ± 0.99 ^c^	56.94 ± 2.01 ^a^
16	124.4 ± 2.1 ^b^	−28.9 ± 1.5 ^b c^	0.199 ± 0.016 ^a^	22.97 ± 0.23 ^b^	45.88 ± 2.71 ^b^
22	120.1 ± 0.1 ^c^	−27.1 ± 1.3 ^c^	0.213 ± 0.009 ^a^	27.76 ± 0.06 ^a^	31.52 ± 1.97 ^c^
28	113.3 ± 0.4 ^d^	−24.1 ± 1.1 ^d^	0.210 ± 0.009 ^a^	3.50 ± 0.04 ^e^	32.85 ± 2.58 ^c^

The values shown represent the mean ± SD. ^a–e^: Different letters in the same column represent significant differences (*p* < 0.05).

## Data Availability

The original contributions presented in the study are included in the article, further inquiries can be directed to the corresponding author.
